# Expression of matrix metalloproteinases and their inhibitors in corneal stromal fibroblasts and keratocytes from healthy and keratoconus corneas

**DOI:** 10.1007/s00417-024-06601-y

**Published:** 2024-08-23

**Authors:** Tim Berger, Elias Flockerzi, Maximilian Berger, Ning Chai, Tanja Stachon, Nóra Szentmáry, Berthold Seitz

**Affiliations:** 1 Department of Ophthalmology, Saarland University Medical Center, Homburg/Saar, Germany; 2 Dr. Rolf M. Schwiete Center for Limbal Stem Cell and Congenital Aniridia Research, Saarland University, Homburg/Saar, Germany

**Keywords:** Keratoconus, Corneal fibroblasts, Keratocytes, Matrix metalloproteinase, Tissue inhibitor of metalloproteinase

## Abstract

**Purpose:**

To examine the in-vitro expression of matrix metalloproteinases (MMP) and tissue inhibitors of metalloproteinases (TIMP) in corneal stromal cells by distinguishing between fibroblasts and keratocytes of healthy and keratoconus (KC) corneas.

**Methods:**

Stromal cells were isolated from healthy and KC corneas (*n* = 8). A normal-glucose, serum-containing cell culture medium (NGSC-medium) was used for cultivation of healthy human corneal fibroblasts (HCFs) and KC human corneal fibroblasts (KC-HCFs). In order to obtain a keratocyte phenotype, the initial cultivation with NGSC-medium was changed to a low-glucose, serum-free cell culture medium for healthy (Keratocytes) and KC cells (KC-Keratocytes). Gene and protein expression of MMP-1, -2, -3, -7, -9 and TIMP-1, -2, -3 were measured by quantitative PCR and Enzyme-Linked Immunosorbent Assay (ELISA) from the cell culture supernatant.

**Results:**

KC-HCFs demonstrated a lower mRNA gene expression for MMP-2 compared to HCFs. In contrast to their respective fibroblast groups (either HCFs or KC-HCFs), Keratocytes showed a higher mRNA gene expression of TIMP-3, whereas TIMP-1 mRNA gene expression was lower in Keratocytes and KC-Keratocytes. Protein analysis of the cell culture supernatant revealed lower concentrations of MMP-1 in KC-HCFs compared to HCFs. Compared to Keratocytes, TIMP-1 concentrations was lower in the cell culture supernatant of KC-Keratocytes. In HCFs and KC-HCFs, protein levels of MMP-1 and TIMP-1 were higher and MMP-2 was lower compared to Keratocytes and KC-Keratocytes, respectively.

**Conclusion:**

This study indicates an imbalance in MMP and TIMP expression between healthy and diseased cells. Furthermore, differences in the expression of MMPs and TIMPs exist between corneal fibroblasts and keratocytes, which could influence the specific proteolytic metabolism in-vivo and contribute to the progression of KC.

**Supplementary Information:**

The online version contains supplementary material available at 10.1007/s00417-024-06601-y.

## Key messages

*****What is known***:**

• Keratoconus (KC) is characterized by an imbalance between matrix metalloproteinases (MMPs) and their inhibitors (tissue inhibitors of metalloproteinases, TIMPs), which can lead to increased proteolytic activity and further progression of the disease.

*****What is new***:**

• Expression differences of various MMPs and TIMPs were found between corneal fibroblasts and keratocytes, emphasizing the importance of cell culture conditions for the maintenance of cell phenotype.
• Although several MMPs and TIMPs were found to differ at the gene and protein level between healthy and KC cells, even well-studied MMPs such as MMP-9, which has been described as altered in KC tear samples, were not expressed in stromal cells, raising questions about epithelial-stromal interactions in KC.

**Supplementary Information:**

The online version contains supplementary material available at 10.1007/s00417-024-06601-y.

## Introduction

Keratoconus (KC) is a bilateral progressive corneal ectasia, which causes conical protrusion and apical thinning resulting in irregular astigmatism and visual distortion [1–3].

The mechanisms involved in the development and progression of KC are still unknown, but a multifactorial process is generally assumed [1, 2, 4]. Although KC is typically classified as a non-inflammatory condition, there is evidence of an inflammatory component, which could induce increased degradation of the extracellular matrix (ECM) [5–8]. Kao et al. were the first to report increased collagenase and gelatinase activities in KC corneas more than 40 years ago and concluded that KC may represent a collagenolytic disease [9].

In recent years, considerable attention has been paid to understanding the role of matrix metalloproteinases (MMP) and tissue inhibitors of metalloproteinases (TIMP) in the pathogenesis of KC [10, 11]. MMPs are a group of zinc-dependent endopeptidases that remodel the ECM, and have the potential to degrade almost any component of the ECM. These enzymes are regulated by endogenous protein inhibitors known as TIMPs [12].

There is evidence that KC patients might have a higher proteolytic activity, as indicated by increased levels of MMPs or collagen degradation products in tear samples and increased MMP expression in epithelial and stromal corneal cells [10, 11, 13–17]. MMPs that have been associated with higher protein levels in tear samples of KC patients include MMP-1, -3, -7 and -9 [6, 13, 18]. Some of the investigations are contradictory in the literature, such as for MMP-2, which showed increased activity in one study [17] but not in another [19]. In contrast, TIMPs as important regulators of MMPs demonstrated a downregulation of TIMP-1, -2 and -3 gene expression [20, 21]. However, the majority of studies focused on tear sample measurements.

Therefore, the purpose of this study was to examine the expression of MMPs and TIMPs in-vitro to identify expression differences between stromal cells from healthy and KC corneas regarding the hypothesis of increased activity of proteolytic enzymes in KC. Another objective of this study was to compare the expression levels by distinguishing between corneal fibroblasts and keratocytes. As KC corneas have a lower keratocyte density with imbalance between keratocytes and fibroblasts, cellular differentiation might provide a better insight into the enzymatic activity in-vivo [22, 23].

## Materials and methods

### Ethics approval and consent to participate

This study was approved by the Ethics Committee of Saarland/Germany (No. 54/23). The Declaration of Helsinki was respected. Prior to the study, all patients with KC agreed to participate.

### Cell culture

The cell cultivation and subsequent experiments were conducted at the biological laboratory of the Department of Ophthalmology, Saarland University Medical Center in Homburg/Saar, Germany.

Healthy corneas that were used for Descemet membrane endothelial keratoplasty were provided by the LIONS Cornea Bank Saar-Lor-Lux, Trier/Westpfalz following tissue preparation, whereas KC corneal buttons (diameter of 8.0 mm) were obtained from elective penetrating keratoplasties. Descriptive information of KC samples are provided in Table 1 [24–26]. Cells were isolated from eight healthy human corneas (mean donor age: 79 ± 7 years, range: 62–86 years, 50% male, 50% female) and eight KC patients (mean age 43 ± 19 years, range: 20–71 years, 50% male, 50% female).

**Table 1:** Descriptive data of the keratoconus patients including the keratoconus ABCD grading system with the Homburg Biomechanical E-staging [24–26]

Patient number	Patient age	Gender	Keratoconus ABCD grading system with Homburg Biomechanical E-Staging	History of atopy
1	20	Male	A4 B4 C4 D2 E4 +	-
2	23	Male	A4 B4 C3 D3 E4 ++	-
3	29	Female	A4 B4 C4 D3 E4 +	-
4	31	Female	A4 B4 C3 D4 E4 -	-
5	51	Male	A4 B4 C4 D4 E4 ++	-
6	57	Female	A4 B4 C2 D2 E4 +	Mild atopic dermatitis
7	63	Female	A4 B4 C3 D4 E3 +	-
8	71	Male	A2 B3 C0 D2 E2 -	-

Cultivation of corneal stromal cells was initially performed using a normal-glucose, serum-containing cell culture medium (NGSC-medium) that consisted of Dulbecco’s modified Eagle’s medium (DMEM/F12) (Cat-No.: 11320033, Thermo Fisher Scientific, Waltham, MA, USA), 5% fetal calf serum (FCS) (Thermo Fisher Scientific, Waltham, MA, USA), and 1% penicillin-streptomycin (P/S) (Sigma-Aldrich, St. Louis, MO, USA), which is a common method for fibroblast cell culture work [27].

Cell cultivation was either continued with the NGSC-medium (for the fibroblast phenotype) or changed to a low-glucose, serum-free cell culture medium (LGSF-medium) after 2 days and a cell confluence of approximately 20%, which consisted of serum-free low-glucose DMEM (Sigma-Aldrich, St. Louis, MO, USA, Catalog number: D6046) with 1 mM L-ascorbic acid, 2 g/l D-glucose, 2.5 g/l D-mannitol, 1% insulin-transferrin-sodium selenite (ITS, Sigma-Aldrich, St. Louis, MO, USA, Catalog number: 1884) and 1% P/S [28].

Cell cultivation was performed using either NGSC-medium for the fibroblast phenotype or LGSF-medium for the keratocyte phenotype, which was changed every 5 days until complete confluence for keratocytes (average cell culture time of 14 days) and for fibroblasts (average cell culture time of 6 days) was achieved. Therefore, measurements were conducted on healthy human corneal fibroblasts (HCFs), KC human corneal fibroblasts (KC-HCFs), healthy keratocyte cells (Keratocytes) and KC keratocyte cells (KC-Keratocytes).

Culture medium of corneal fibroblasts and keratocytes was replaced 48 h before harvesting the cells. The cell pellet and culture supernatant were frozen at -80 °C until further use.

### RNA isolation and cDNA synthesis

RNA isolation was performed following the manufacturer’s instructions (Total RNA Purification Plus Micro Kit, Norgen Biotek, Thorold, Canada, Catalog number: 48500) and is based on the principle of spin column chromatography described by Boom et al. [29]. The RNA concentration was determined at a wavelength of 260 nm using a spectrophotometer (ScanDrop 100 Analytik Jena GmbH & Co. KG, Jena, Germany) in a 1:10 dilution with nuclease-free water. A blank of 50 µl nuclease-free water was used. The isolated RNA was checked for purity using the quotients of A260/A230 and A260/A280 and stored at -80 °C until further use.

The complementary deoxyribonucleic acid (cDNA), which is required as a template for qPCR, was synthesized according to Gubler et al. [30]. The One Taq^®^ RT-PCR Kit (New England Biolabs Inc., Frankfurt, Germany, Catalog number: E5310S) was used for cDNA synthesis, which required 1 µg total RNA for all samples and was calculated according to the previously spectrophotometrically determined concentration. First, RNA and oligo-dT primers, which ensure selective amplification of mRNA, were incubated for 5 min at 70 °C in the MiniAmp thermocycler to allow denaturation of the RNA with primer attachment. The provided components M-MuLV Enzyme Mix and M-MuLV Reaction Mix were then added to the RNA preparation and incubated at 42 °C for one hour. The reverse transcriptase was inactivated by heating at 80 °C for 5 min, the synthesized cDNA was diluted with 30 µl nuclease-free water to a final volume of 50 µl and stored at -20 °C.

### Quantitative PCR

The quantitative Polymerase Chain Reaction (qPCR) mix (total volume: 9 µl) contained 1 µl of the specific primer solution, 5 µl SYBR Green Mix (Vazyme Biotech Co, Nanjing, China), and 3 µl nuclease-free water. qPCR was performed using the QuantStudio 5 real-time PCR system (Thermo Fisher Scientific, Waltham, MA, USA) and samples were run in 9 µl volume with 1.5 µl cDNA according to the manufacturer’s instructions. The quantification of the amplified double-stranded DNA is based on the measurement of the signal intensity emitted by the fluorescence dye SYBR Green [31]. The amplification conditions (40 cycles) were 95 °C for 10 s (denaturation), 60 °C for 30 s (primer hybridization), and 95 °C for 15 s (elongation). Every sample was measured in duplicate. CT values were normalized to TATA-binding protein (TBP) as an endogenous reference gene using the ΔCT method and the fold change (2^−ΔΔCT−value^) was used for statistical analysis. The following primers were used for qPCR: CD34, collagen 5, keratocan, lumican, MMP-1, -2, -3, -7, -9, TBP, TIMP-1, -2, -3. Further information about the primers are provided in the Supplementary Table 1. Healthy HCFs served as reference (fold change = 1).

### Protein quantification

Cells of a confluent 75-cm^2^ culture flask were lysed with RIPA buffer (Thermo Fisher Scientific, Waltham, MA, USA) followed by determining the protein concentration with the Pierce™ BCA Protein Assay Kit (Thermo Fisher Scientific, Waltham, MA, USA, Catalog number: 23225), using dilution series of bovine serum albumin for standard curve generation. The method is based on the reduction of Cu^2+^ ions to Cu^1+^ ions in the presence of proteins in an alkaline environment. The complex formation of Cu^1+^ ions with bicinchoninic acid (BCA) leads to a color change, the intensity of which could be measured photometrically [32]. Duplicate measurements were performed with the Tecan Infinite F50 Absorbance Microplate Reader (Tecan Group AG, Männedorf, Switzerland) at a wavelength of 560 nm.

### Enzyme-linked immunosorbent assay (ELISA)

Enzyme-linked Immunosorbent Assay (ELISA) was performed to determine the concentration of MMPs (MMP-1, -2, -3, -9) and TIMPs (TIMP-1, -2, -3) in the cell culture supernatant of eight independent samples of healthy and KC stromal cells.

The detection procedure was based on the principle of the sandwich ELISA technique using a specific ELISA Kit (Supplementary Table 2), according to the provided instructions [33]. Generally, 100 µl of the capture antibody was added to each well of the plate, followed by overnight incubation at room temperature. Subsequently, 100 µl of the collected supernatant was added to the respective wells and incubated for 2 h. The detection antibody was then added and incubated for a further 2 h. The concentrations of the respective MMPs and TIMPs were quantified using recombinant human protein as a standard.

Photometric measurements were performed in duplicate with 100 µl cell culture supernatant by the Tecan Infinite F50 Absorbance Microplate Reader using a provided standard curve.

Measured concentrations of the specific proteins in the cell culture supernatant were divided by the total protein concentrations of the cell lysate to obtain the respective concentration in picogram per milligram of total protein. The quotient (pg/mg of total protein) was used for further statistical analysis.

### Statistical analysis

GraphPad Prism 9.0.0 (GraphPad Software, Boston, MA, USA) was used for statistical analysis. Data were expressed as mean ± standard deviation (SD). There were no missing data in this study and each group had 8 valid measurements of gene and protein expression. Results were checked for normality using the Shapiro-Wilk test, which confirmed a normal distribution. Measurements between two groups were analyzed by an unpaired t-test. Measurements among more than two groups (multiple comparison) were analyzed using a one-way ANOVA and Bonferroni post hoc analysis. P-values < 0.05 were considered statistically significant.

## Results

### Cell type confirmation

Human corneal stromal cells cultured in LGSF- and NGSC-medium demonstrated differences in cell morphology. Cells cultured in NGSC-medium (HCFs / KC-HCFs) showed a fusiform shape typical for fibroblasts, whereas cells cultured in LGSF-medium (Keratocytes / KC-Keratocytes) exhibited a dendritic cell type indicative of a keratocyte phenotype. Microscopic images of the different phenotypes have been previously published [8]. Gene expression of keratocyte-specific markers such as collagen 5 (*p* < 0.0001 / *p* = 0.0001), CD34 (*p* = 0.0073 / *p* = 0.0001), keratocan (*p* = 0.0336 / *p* = 0.0196) and lumican (*p* = 0.0004 / *p* = 0.0006) was higher in Keratocytes and KC-Keratocytes compared to HCFs or KC-HCFs.

### Gene expression analysis

The mRNA gene expression results are provided in Tables 2 and 3.

**Table 2:** Gene expression (mRNA) of matrix metalloproteinases (MMP) and tissue inhibitors of metalloproteinases (TIMP) in healthy human corneal fibroblasts (HCFs), keratoconus human corneal fibroblasts (KC-HCFs), healthy human keratocytes (Keratocytes), and keratoconus human keratocytes (KC-Keratocytes)

Gene	Group relative expression - Fold change (2^−ΔΔCT^)			
	HCFs	KC-HCFs	Keratocytes	KC-Keratocytes
MMP-1	1.00 ± 0.00	0.93 ± 0.88	7.77 ± 6.70	8.55 ± 7.64
MMP-2	1.00 ± 0.00	0.59 ± 0.36	0.96 ± 0.56	0.67 ± 0.15
MMP-3	1.00 ± 0.00	0.61 ± 0.39	5.34 ± 5.77	2.30 ± 3.75
TIMP-1	1.00 ± 0.00	0.96 ± 0.25	0.54 ± 0.27	0.46 ± 0.26
TIMP-2	1.00 ± 0.00	0.76 ± 0.61	2.16 ± 1.71	1.73 ± 1.81
TIMP-3	1.00 ± 0.00	0.73 ± 0.58	2.40 ± 1.44	2.11 ± 1.43

The following genes were measured: MMP-1, MMP-2, MMP-3, TIMP-1, TIMP-2 and TIMP-3. Fold changes are expressed as mean ± SD of eight independent samples of healthy donor or KC corneas

**Table 3:** *P*-values for multiple group comparisons of mRNA gene expression of matrix metalloproteinases (MMP-1, MMP-2, MMP-3) and tissue inhibitors of metalloproteinases (TIMP-1, TIMP-2, TIMP-3) between healthy human corneal fibroblasts (HCFs), keratoconus human corneal fibroblasts (KC-HCFs), healthy human keratocytes (Keratocytes), and keratoconus human keratocytes (KC-Keratocytes)

Gene	*p*-value (HCFs vs. KC-HCFs)	*p*-value (Keratocytes vs. KC-Keratocytes)	*p*-value (HCFs vs. Keratocytes)	*p*-value (KC-HCFs vs. KC-Keratocytes)
MMP-1	> 0.9999	0.9930	0.0727	0.0890
MMP-2	**0.0428**	0.4508	0.9974	0.9662
MMP-3	0.9964	0.3692	0.0987	0.7970
TIMP-1	0.9930	0.9047	**0.0024**	**0.0021**
TIMP-2	0.9818	0.9095	0.2880	0.4372
TIMP-3	0.9556	0.9527	**0.0459**	0.0522

Significant p-values < 0.05 were highlighted in bold font

MMP-7 and  -9 mRNA gene expression was not detectable in corneal fibroblasts (HCFs / KC-HCFs) and keratocytes (Keratocytes / KC-Keratocytes). In KC-HCFs, mRNA gene expression was lower for MMP-2 (*p* = 0.0428) in contrast to HCFs. Compared to the respective fibroblast group (HCFs or KC-HCFs), the mRNA gene expression of TIMP-3 (*p* = 0.0459) was higher in Keratocytes, while the mRNA gene expression of TIMP-1 was lower in Keratocytes (*p* = 0.0024) and KC-Keratocytes (*p* = 0.0021).

### Protein expression analysis of the cell culture supernatant (ELISA)

The protein expression results are provided in Tables 4 and 5.

**Table 4:** Protein expression of matrix metalloproteinases (MMP) and tissue inhibitors of metalloproteinases (TIMP) of the cell culture supernatant in healthy human corneal fibroblasts (HCFs), keratoconus human corneal fibroblasts (KC-HCFs), healthy human keratocytes (Keratocytes) and keratoconus human keratocytes (KC-Keratocytes)

Protein	Group (pg/mg of total protein)			
	HCFs	KC-HCFs	Keratocytes	KC-Keratocytes
MMP-1	1080.0 ± 249.0	688.5 ± 292.7	498.7 ± 309.0	215.6 ± 266.5
MMP-2	13700 ± 5300	11200 ± 3200	26550 ± 11500	23200 ± 10600
MMP-3	2089 ± 1229	1368 ± 651.6	1401 ± 1344	297.1 ± 396.2
TIMP-1	65390 ± 5076	56961 ± 7399	34139 ± 10015	23283 ± 5483
TIMP-2	12365 ± 2902	12486 ± 2311	14456 ± 5621	13357 ± 3238

The following proteins were measured: MMP-1, MMP-2, MMP-3, TIMP-1 and TIMP-2. Measured concentrations of the specific proteins in the cell culture supernatant were divided by the total protein concentrations of the cell lysate to obtain the respective concentration in picogram per milligram of total protein. Data are expressed as mean ± SD comprising eight independent samples of healthy donors or KC corneas

**Table 5:** P-values for multiple group comparisons of protein expression of matrix metalloproteinases (MMP-1, MMP-2, MMP-3) and tissue inhibitors of metalloproteinases (TIMP-1, TIMP-2) of the cell culture supernatant between healthy human corneal fibroblasts (HCFs), keratoconus human corneal fibroblasts (KC-HCFs), healthy human keratocytes (Keratocytes), and keratoconus human keratocytes (KC-Keratocytes)

Protein	*p*-value (HCFs vs. KC-HCFs)	*p*-value (Keratocytes vs. KC-Keratocytes)	*p*-value (HCFs vs. Keratocytes)	*p*-value (KC-HCFs vs. KC-Keratocytes)
MMP-1	**0.0493**	0.3211	**0.0058**	**0.0284**
MMP-2	0.9322	0.8661	**0.0256**	**0.0384**
MMP-3	0.4746	0.2396	0.5767	0.2126
TIMP-1	0.1397	**0.0454**	**< 0.0001**	**< 0.0001**
TIMP-2	0.9999	0.9366	0.6881	0.9634

Significant *p*-values < 0.05 were highlighted in bold font

The protein concentration levels of MMP-9 and TIMP-3 in the cell culture supernatant were below the detection limit. In KC-HCFs, the MMP-1 (*p* = 0.0493) protein concentration in the cell culture supernatant was lower than in HCFs. TIMP-1 (*p* = 0.0454) protein concentration in the cell culture supernatant was higher in Keratocytes than in KC-Keratocytes. In HCFs and KC-HCFs, protein levels of MMP-1 (*p* = 0.0058 / *p* = 0.0284) and TIMP-1 were higher (*p* < 0.0001 / *p* < 0.0001) and MMP-2 was lower (*p* = 0.0256/ 0.0384) in contrast to Keratocytes and KC-Keratocytes, respectively.

## Discussion

Increased proteolytic activity has been hypothesized to be one of the major contributing factors in progressive thinning of KC corneas since it was first reported in 1982 [9].

One of the main group of enzymes associated with increased proteolytic activity are MMPs, which are involved in physiological processes such as tissue remodeling, wound healing, inflammation, angiogenesis and embryonic development [34–36]. MMPs can cleave and degrade ECM proteins, however it is estimated that only about 31% of their substrates are ECM proteins and around 69% are non-ECM proteins [35, 37]. Each of these enzymes has a specificity for different and often overlapping substrates and is modulated by transcriptional and post-transcriptional mechanisms, proteolytic activation, post-translational modifications and extracellular inhibition [36, 38]. The activity of mature MMPs is regulated by four different forms of TIMPs [36]. Under homeostatic conditions, most MMPs are not produced or only produced at low amounts. However, when cells are stimulated with cytokines or growth factors, MMP production increases [35]. Therefore, it is evident that the disturbance of these regulatory mechanisms may result in excessive MMP activity and pathological degradation of the ECM [39].

Because of its easy sample collection, the tear film is an adequate medium for investigating different ocular diseases. However, the human tear film is a highly variable construct with large fluctuations that can be altered by various ocular diseases, for example, dry eye disease, which can lead to increased cytokine and MMP concentrations in the tear fluid [40, 41]. To date, several studies have analyzed MMP concentrations in KC patients. It has been shown that tear samples from patients with KC have higher MMP-1, -3, -7 and − 13 protein levels as well as a higher gelatinolytic and collagenolytic activity compared to normal subjects [13]. Lema et al. observed an overexpression of Interleukin-6 (IL-6), Tumor necrosis factor α (TNF-α) and MMP-9 in tear samples of KC patients and concluded that a chronic inflammatory activity might be relevant in the pathogenesis of KC [6]. While IL-6 and TNF-α protein concentrations were higher in tear samples of subclinical and manifest KC eyes, MMP-9 was only elevated in the latter group [18]. Similarly, Shetty et al. have described increased IL-6 and MMP-9 levels in tear samples of patients with KC [15]. Balasubramanian et al. demonstrated that increased eye rubbing led to higher IL-6, TNF-α and MMP-13 protein levels in tear samples from healthy subjects, although collagenolytic activity did not change. They assumed that prolonged and intense eye rubbing, as frequently observed in patients with KC, could therefore contribute to the progression of the disease [14]. In addition to an altered MMP protein expression in tear samples, increased levels of collagen degradation products such as telopeptides have been described in KC patients, supporting the hypothesis of increased proteolytic activity [42]. The precise origin of the elevated MMP protein levels in KC tear samples is still unclear, as they may originate from secretory active cells such as epithelial cells of the cornea or conjunctiva, or other cell types. Based on an altered MMP metabolism in tear samples of KC patients indicating a higher proteolytic activity, this study analyzed a variety of different MMPs and TIMPs on gene and protein level in corneal stromal cells. It has been demonstrated that KC-HCFs had a lower MMP-2 mRNA gene expression than HCFs. Furthermore, the MMP-1 protein level was lower in the cell culture supernatant of KC-HCFs.

Another purpose of this study was to compare different stromal cell types, namely keratocytes and corneal fibroblasts, by using two cell culture media compositions. It is known that stromal cells that are cultured in a cell culture medium containing FCS and relatively high concentrations of glucose do not retain their keratocyte-specific phenotype and transform into corneal fibroblasts in-vitro. However, the keratocyte-specific phenotype can be preserved by using a glucose-reduced, serum-free cell culture medium, providing a better opportunity to study cellular behavior in KC closer to in-vivo conditions [28]. The comparison of keratocytes and fibroblasts in KC corneas could offer a different perspective with regard to potential proteolytic changes, as the matrix-synthetic phenotype of fibroblasts is quite different from that of keratocytes. Additionally, as KC corneas have a lower keratocyte density with imbalance between keratocytes and fibroblasts, cellular differentiation might provide a better insight into the enzymatic activity in-vivo [22, 23]. No differences in mRNA gene expression between healthy Keratocytes and KC-Keratocytes were detected in this study. However, the TIMP-1 protein level in the cell culture supernatant of KC-Keratocytes was lower compared to healthy Keratocytes.

Kenney et al. described a higher gelatinolytic activity in the supernatant of KC-Keratocytes compared to that of healthy Keratocytes. However, zymography showed identical enzyme patterns for MMP-2 (pro-form and the activated form) between healthy and KC-Keratocytes, leading the authors to assume that the increased gelatinolytic activity in KC probably does not correlate with an increased amount of activated MMP-2 [19]. Interestingly, reduced TIMP protein levels were measured in the cell culture supernatant of KC-Keratocytes, with a threefold increase in the MMP-2/TIMP ratio, which might play a significant role in the higher gelatinolytic activity [19].

In this study, several differences between corneal fibroblasts and keratocytes were detected at gene and protein levels. Compared to the respective fibroblast group, Keratocytes showed an increased TIMP-3 mRNA gene expression, whereas TIMP-1 mRNA gene expression was decreased in Keratocytes and KC-Keratocytes. In the cell culture supernatant of Keratocytes and KC-Keratocytes, MMP-1 and TIMP-1 were lower and MMP-2 higher than in the corresponding fibroblast group.

These results suggest that the expression of different MMPs is fundamentally altered between fibroblasts and keratocytes and that an unequal distribution of both cell types might also influence the specific proteolytic metabolism in-vivo.

Furthermore, MMP-9 expression could not be measured in corneal stromal cells, neither in fibroblasts nor in keratocytes, as the gene and protein expression levels were too low. This appears unexpected, as MMP-9 is one of the most studied MMPs in the tear film of KC patients, but is barely expressed by stromal cells in-vitro. In contrast to the findings of the present study demonstrating no upregulation of MMPs in stromal KC cells, epithelial cells from KC patients showed increased TNF-α, IL-6 and MMP-9 gene expression [15]. Additionally, it is noteworthy that the MMP-9 mRNA gene expression of epithelial cells in-vitro and the MMP-9 protein concentration in tear samples of KC patients decreased after treatment with cyclosporine A, indicating an inflammatory relationship [15]. In a study by Predović et al., MMP-9 protein concentrations were found to be higher in the epithelium than in the corneal stroma of KC patients. However, the stromal MMP-9 concentration did not vary between patients with KC and bullous keratopathy [43]. The expression of MMP-9 also seems to differ within the same corneal layer, as a higher mRNA expression was found at the cone apex than in the peripheral cornea for both the epithelium and the stroma, but no comparison was made at the protein level in this study [44]. The lack of MMP-9 expression in the present study might be attributed to a low baseline expression in stromal cells, differences between in-vitro and in-vivo expression or the cell culture conditions, among other factors.

Matthews et al. reported that an imbalance of TIMP-1 and − 3 may promote the keratocyte apoptosis in KC, which is the most common form of cell death in this disease [45]. An immunohistochemical study examined TIMP-1, -2 and -3 as well as MMP-2 and MMP-9 staining in healthy and KC corneas. There were no distinct differences between healthy and diseased corneas and the immunostaining was generally most evident in the corneal epithelium [46].

Despite many years of research, the precise role of MMPs and TIMPs in KC is not clearly understood. Because KC is a slowly progressive corneal ectasia, only temporary changes in the activity of these enzymes might be sufficient to weaken the corneal stroma. Various mechanisms (eye rubbing, UV radiation, contact lenses, allergies) probably contribute to the development of this disease in people who may be susceptible [11]. We were able to show an altered expression of various MMPs and TIMPs between healthy and diseased cells, as well as an altered expression between fibroblasts and keratocytes. However, it should be noted, that one of the limitations of this study is the small sample size of eight, which might change the results statistically if the number of samples were increased. Nevertheless, the in-vivo mechanisms are much more complex and especially the interaction between epithelium and stroma as a potential driver of proteolytic degradation might play a greater role than previously assumed.

## Electronic supplementary material

Below is the link to the electronic supplementary material.

Supplementary Material 1
