# Supplementary material for: Expression of matrix metalloproteinases and their inhibitors in corneal stromal fibroblasts and keratocytes from healthy and keratoconus corneas
Source: Graefes Arch Clin Exp Ophthalmol. 2024 Aug 23;263(2):467–75. doi: 10.1007/s00417-024-06601-y (PMC11868159; doi:10.1007/s00417-024-06601-y)
Supplement: Supplementary file 1 — Supplementary Material 1 [file 417_2024_6601_MOESM1_ESM.docx]

**Supplementary material**

**Title:** Expression of matrix metalloproteinases and their inhibitors in corneal stromal fibroblasts and keratocytes from healthy and keratoconus corneas

**Journal name:** Graefe's Archive for Clinical and Experimental Ophthalmology

**Authors:**

Tim Berger, MD ^1^, Elias Flockerzi, MD ^1^, Maximilian Berger, MD ^1^, Ning Chai, MD ^2^, Tanja Stachon, M.Sc. ^2^, Nóra Szentmáry, MD, PhD, DSc ^2^, Berthold Seitz, MD ^1^

^1^ Department of Ophthalmology, Saarland University Medical Center, Homburg/Saar, Germany

^2^ Dr. Rolf M. Schwiete Center for Limbal Stem Cell and Congenital Aniridia Research, Saarland University, Homburg/Saar, Germany

**Corresponding author:**

Dr. Tim Berger, MD

Department of Ophthalmology**,** Saarland University Medical Center, Kirrberger Str., D-66424 Homburg/Saar, Germany

Telephone number: 0049 6841 16-22302

Fax number: 0049 6841 16-22479

E-mail: tim.berger@uks.eu

| **Primer** | **QIAGEN catalog number** | **Manufacturer (company, city, country)** |
| --- | --- | --- |
| CD34 | QT00056497 | Qiagen N.V., Venlo, Netherlands |
| Collagen 5 | QT00217392 |  |
| Keratocan | QT00021280 |  |
| Lumican | QT00058982 |  |
| Matrix metalloproteinase 1 (MMP-1) | QT00014581 |  |
| Matrix metalloproteinase 2 (MMP-2) | QT00088396 |  |
| Matrix metalloproteinase 3 (MMP-3) | QT00060025 |  |
| Matrix metalloproteinase 7 (MMP-7) | QT00001456 |  |
| Matrix metalloproteinase 9 (MMP-9) | QT00040040 |  |
| TATA-binding protein (TBP) | QT00000721 |  |
| Tissue Inhibitor of metalloproteinase 1 (TIMP-1) | QT00084168 |  |
| Tissue Inhibitor of metalloproteinase 2 (TIMP-2) | QT00017759 |  |
| Tissue Inhibitor of metalloproteinase 3 (TIMP-3) | QT00046382 |  |

**Supplementary Table 1:** Primer pairs used for quantitative Polymerase Chain Reaction (qPCR).

| **ELISA Kit** | **Catalog number** | **Assay range** | **Manufacturer (company, city, country)** |
| --- | --- | --- | --- |
| Human Total MMP-1 DuoSet ELISA | DY901B | 62.5 – 4000 pg/ml | R&D Systems, Minneapolis, MN, USA |
| Human MMP-2 DuoSet ELISA | DY902 | 0.6 – 20 ng/ml |  |
| Human Total MMP-3 DuoSet ELISA | DY513 | 31.2 – 2000 pg/ml |  |
| Human MMP-9 DuoSet ELISA | DY911 | 31.2 – 2000 pg/ml |  |
| Human TIMP-1 DuoSet ELISA | DY970 | 31.2 – 2000 pg/ml |  |
| Human TIMP-2 DuoSet ELISA | DY971 | 31.2 – 2000 pg/ml |  |
| Human TIMP-3 DuoSeat ELISA | DY973 | 62.5 – 4000 pg/ml |  |

**Supplementary Table 2:** Enzyme-linked Immunosorbent Assay (ELISA) kits used for determination of protein concentration in cell culture supernatant.
